# Two new species of Lecithoceridae (Lepidoptera, Gelechioidea), with a revised check list of the family in Taiwan

**DOI:** 10.3897/zookeys.263.3781

**Published:** 2013-02-04

**Authors:** Kyu-Tek Park, John B. Heppner, Yang-Seop Bae

**Affiliations:** 1 McGuire Center for Lepidoptera and Biodiversity, Florida Museum of the Natural History, University of Florida, Gainesville, FL 32611 USA; 2 Division of Life Sciences, College of Life Sciences and Bioengineering, University of Incheon, Incheon, 406-772 Korea

**Keywords:** *Caveana*, *Lecithocera*, Lepidoptera, Lecithoceridae, new species, Taiwan, taxonomy

## Abstract

Two species of Lecithoceridae (Lepidoptera, Gelechioidea), *Caveana senuri* **sp. n.** and *Lecithocera dondavisi* **sp. n.**, are described from Taiwan. The monotypic *Caveana* Park was described from Thailand, based on *Caveana diemseoki* Park, 2011. *Lecithocera* Herrich-Schäffer, 1853 is the most diverse genus of the family, comprising more than 300 species worldwide. *Lecithocera dondavisi* **sp. n.** is the largest species of the genus so far, and closely resembles the Indian species, *Lecithocera praeses* Meyrick, 1919. A revised check list of the family in Taiwan is provided.

## Introduction

The family Lecithoceridae (Lepidoptera, Gelechioidea) is a relatively poorly known group of microlepidoptera that comprises more than 1,200 extant known species worldwide (van Nieukerken et al. 2011). The group has not been well studied, due to the lack of specialists and the relative unattractiveness of the moths due to the larval feeding habitat on debris and being economically irrelevant. The known species of Lecithoceridae are mostly restricted to the Oriental and Australian regions, but the distributional range includes the southern part of the Palaearctic Region.

In Taiwan, a total of 63 species of Lecithoceridae have been reported (Park 2000a; Park and Wang 2000; Park 2003a, b), with 30 species known as endemic. *Lecithocera* Herrich-Schäffer, 1853 is the most diverse genus of the family with more than 300 species described worldwide, and it is highly diverse in the Oriental Region. The genus is characterized by the presence of M_2_ in the hindwing, the abdomen without spinose zones on tergites, with a bundle of long coremata in abdominal segment VII, and the male genitalia with well-developed costal bar. *Lecithocera* in Taiwan was first reviewed by Park (1999), reporting 22 species with 13 new described species. Park and Wang (2000) and Heppner (2012) enumerated 25 species, including an erroneously cited *Lecithocera theconoma* Meyrick, 1926 (thisspecies was described from Sarawak). In this paper, *Lecithocera dondavisi* Park, sp. n. , the largest species of *Lecithocera*, is described.

The monotypic *Caveana* Park, 2010 was described from Thailand, based on *Caveana diemseoki* Park, 2010. The genus is placed in the subfamily Torodorinae because it shares the presence of spinose zones on the abdominal tergites and the male genitalia lacks costal bars that connects the tegumen and valva. The genus isallied to *Nosphistica* Meyrick, 1911 and *Philoptilia* Meyrick, 1918by having a similar the venation, with M_3_, CuA_1_ and CuA_2_ on a common stalk in the forewing and M_2_ absent from the hindwing, but *Caveana* differs from them by the brightly colored forewing and the lack of rough scale projections of the hindwing costa and strongly sinuate termen. *Philoptilia* is distinguished by the forewing R_5_, which is absent in *Nosphistica*. Park (2010) noted that *Caveana* has a unique concave region beyond the middle of R_2_ on the ventral forewing surface. While *Caveana senuri* has no such concavity in the forewing, this species is still placed in *Caveana*, because of its brightly colored forewing and the male genitalia which are similar to those of *Caveana diemseoki*. *Caveana* is reported for the first time from Taiwan, describing *Caveana senuri* Park, sp. n. in this paper.

A revised check list of the family Lecithoceridae in Taiwan, with 74 known species, is provided in Table 1.

**Table 1.** Check list of Lecithoceridae in Taiwan.

Species	Type locality	Type depository
*Homaloxestis* Meyrick, 1910		
*Homaloxestis baibaraensis* Park, 1999	Taiwan	USNM
*Homaloxestis cholopis* (Meyrick, 1906) (*Lecithocera*)	Myanmar	BMNH
*Homaloxestis hilaris* Gozmány, 1978	Zhejiang, China	ZFMK
*Homaloxestis myeloxesta* Meyrick, 1932	Taiwan	BMNH
*Lecithocera* Herrich-Schäffer, 1853		
*Lecithocera angustiella* Park, 1999	Taiwan	KNA
*Lecithocera altusana* Park, 1999	Taiwan	KNA
*Lecithocera atricastana* Park, 1999	Taiwan	USNM
*Lecithocera aulias* Meyrick, 1910	Khasi Hills, India	BMNH
*Lecithocera bimaculata* Park, 1999	Taiwan	BMNH
*Lecithocera chartaca* Wu & Liu, 1993	Jiangxi, China	IZAS
*Lecithocera dondavisi* Park, sp. n.	Taiwan	MCUF
*Lecithocera erecta* Meyrick, 1935	Zheijang, China	BMNH
*Lecithocera fascicula* Park, 1999	Taiwan	KNA
*Lecithocera fascinatrix* Meyrick, 1935	Taiwan	BMNH
*Lecithocera fuscosa* Park, 1999	Taiwan	KNA
*Lecithocera glabrata* (Wu & Liu, 1992) (*Quassitagma*)	Jiangxi, China	IZAS
*Lecithocera indigens* (Meyrick, 1914) (*Frisilia*)	Taiwan	DEI
*Lecithocera latiola* Park,1999	Taiwan	KNA
*Lecithocera megalopis* Meyrick, 1916	Philippines	BMNH
*Lecithocera metacausta* Meyrick, 1910	Khasi Hills, India	BMNH
*Lecithocera palingensis* Park, 1999	Taiwan	KNA
*Lecithocera paralevirota* Park, 1999	Taiwan	USNM
*Lecithocera pelomorpha* Meyrick, 1931	Sichuan, China	BMNH
*Lecithocera pulchella* Park, 1999	Taiwan	KNA
*Lecithocera rotundata* Gozmány, 1978	Zhejiang, China	ZFMK
*Lecithocera serena* Gozmány, 1978 (*Sarisophora*)	Shaanxi, China	ZFMK
*Lecithocera shanpinensis* Park, 1999	Taiwan	KNA
*Lecithocera thaiheisana* Park, 1999	Taiwan	USNM
*Lecithocera tienchiensis* Park, 1999	Taiwan	KNA
*Lecitholaxa* Gozmány, 1978		
*Lecitholaxa thiodora* (Meyrick, 1914) (*Lecithocera*)	Taiwan	HNHM
*Frisilia* Walker, 1864		
*Frisilia chinensis* Gozmány, 1978	Sichuan, China	BMNH
*Frisilia cornualis* Park, 2008	Taiwan, Vietnam	KNA
*Frisilia homalistis* Meyrick, 1935	Taiwan	BMNH
*Spatulignatha* Gozmány, 1978		
*Spatulignatha idiogena* Wu, 1994	Fujian, China	IZAS
*Spatulignatha olaxana* W, 1994	Zhejiang, China	IZAS
*Synersaga* Gozmány, 1978		
*Synersaga bleszynskii* (Gozmány, 1978) (*Anaminmnesis*)	Zhejiang, China	ZFMK
*Synersaga caradjai* Gozmány, 1978	Taiwan	MGAB
*Carodista* Meyrick, 1925		
*Carodista cultrata* Park, 2000	Taiwan	MCUF
*Carodista montana* Park, 2000	Taiwan	KNA
*Carodista notolychna* (Meyrick, 1936) (*Homaloxestis*)	Taiwan	BMNH
*Dinochares* Meyrick, 1925		
*Dinochares notolepis* Park, 2000	Taiwan	USNM
*Issikiopteryx* Moriuti, 1973		
*Issikiopteryx zonophaera* (Meyrick, 1935) (*Olbothrepta*)	Zhejiang, China	BMNH
*Issikiopteryx taipingensis* Park, 2003	Taiwan	KNA
*Tisis* Walker, 1864		
*Tisis mesozosta* Meyrick, 1914	Taiwan	DEI
*Nosphistica* Meyrick, 1911		
*Nosphistica bisinuata* Park, 2002	Taiwan	KNA
*Nosphistica fenestrata* (Gozmány, 1978) (*Philoptila*)	Fujian, China	HMNH
*Nosphistica fuscolepis* Park, 2002	Taiwan	USNM
*Nosphistica parameocola* (Wu, 1996) (*Athymoris*)	Hainan, China	IZAS
*Nosphistica tarokoensis* Park, 2002	Taiwan	KNA
Subfamily TORODORINAE		
*Torodora* Meyrick, 1894		
*Torodora albicruris* Park & Heppner, 2000	Taiwan	USNM
*Torodora capillaries* Park & Heppner, 2000	Taiwan	USNM
*Torodora chianensis* Park, 2003	Taiwan	USNM
*Torodora manoconta* Wu & Liu, 1994	Jiangxi, China	IZAS
*Torodora octavana* (Meyrick, 1911)(*Brachmia*)	Khasi Hills, India	BMNH
*Torodora parthenopis* (Meyrick, 1932) (*Lecithocera*)	Taiwan	BMNH
*Torodora pseudogalera* Park, 2003	Taiwan	USNM
*Torodora rectilinea* Park, 2003	Taiwan	MNHU
*Torodora sciadosa* Wu & Liu, 1994	Sichuan, China	IZAS
*Torodora ortilege* (Meyrick, 1911)	Khasi Hills, India	BMNH
*Deltoplastis* Meyrick, 1925		
*Deltoplastis commatopa* Meyrick, 1932	Taiwan	BMNH
*Deltoplastis lobigera* Gozmany, 1978	Zhejiang, China	ZFMK
*Deltoplastis ovatella* Park, 2001	Taiwan	MCUF
*Thubana* Walker, 1864		
*Thubana albisignis* (Meyrick, 1914) (*Lecithocera*)	Taiwan	DEI
*Thubana deltaspis* Meyrick, 1935	Taiwan	BMNH
*Caveana* Park, 2010		
*Caveana senuri* Park, sp. n.	Taiwan	MCFU
*Athymoris* Meyrick, 1935		
*Athymoris aurantiella* Park, 2000	Taiwan	MCUF
*Athymoris liukueiensis* Park, 2000	Taiwan	MCUF
*Athymoris martialis* Meyrick, 1935	Taiwan	BMNH
*Athymoris phreatosa* (Wu, 1994)	Sichuan, China	IZAS
*Athymoris subtrigona* Park, 2000	Taiwan	MCFU
*Halolaguna* Gozmány, 1978		
*Halolaguna oncopteryx* (Wu, 1994)	Sichuan, China	IZAS
*Halolaguna palinensis* Park, 2000	Taiwan	KNA
*Halolaguna sublaxata* Gozmány, 1978	Kiangsu, China	HNMH
*Philharmonia* Gozmány, 1978		
*Philharmonia adusta* Park 2000	Taiwan	MCFU

BMNH- The Natural History Museum, London, UK; HMNH- Hungarian Museum of Natural History, Budapest, Hungary; IZAS- Institute of Zoology, Academia Sinica, Beijing, China; DEI- Deutsches Entomologisches Institut, Eberswald, Germany; KNA- Korea national Arboretum, Pocheon, Korea; MCUF- McGuire Center for Lepidoptera and Biodiversity, University of Florida, Gainesville, USA; MNHU- Museum für Naturkunde, Zentralinstitu Hummboldt-Universität, Berlin, Germany; USNM- U. S. National Museum of Natural History, Washington, USA.

## Material and methods

Most specimens examined were collected in 1980 and 1989 by the second author and H. Wang, researcher in the National Taiwan Museum, Taipei, Taiwan, and Donald R. Davis, US National Museum of Natural History, Smithsonian Institution (USNM), Washington D.C., USA. The material is preserved in the collections of USNM and the McGuire Center for Lepidoptera and Biodiversity, Florida Museum of the Natural History, University of Florida (MCUF), Gainesville, FL, USA. The holotypes of the new species are deposited in MCUF and paratypes are in both museums, on indefinite loan from Taiwan.

## Taxonomic accounts

### Caveana senuri

Park sp. n.

urn:lsid:zoobank.org:act:BB330AA1-77FD-4D94-B7AE-D02781BAD8A9

http://species-id.net/wiki/Caveana_senuri

Figs 1 –12

#### Diagnosis.

The light-orange color pattern of the forewing is unique, with dark-brown streaks between veins. The pattern is more or less similar to that of *Timyra aulonitis* Meyrick, 1908 which was described from Sri Lanka, but the species can be distinguished by the venation of both wings, and by the absence of the scale projection in the basal segment of antenna and the scale-tuft in the hind tibia which are unique to *Timyra* Walker, 1864. The male genitalia are also different from those of the *Timyra aulonitis*.

#### Description.

**Figures 1–5.**
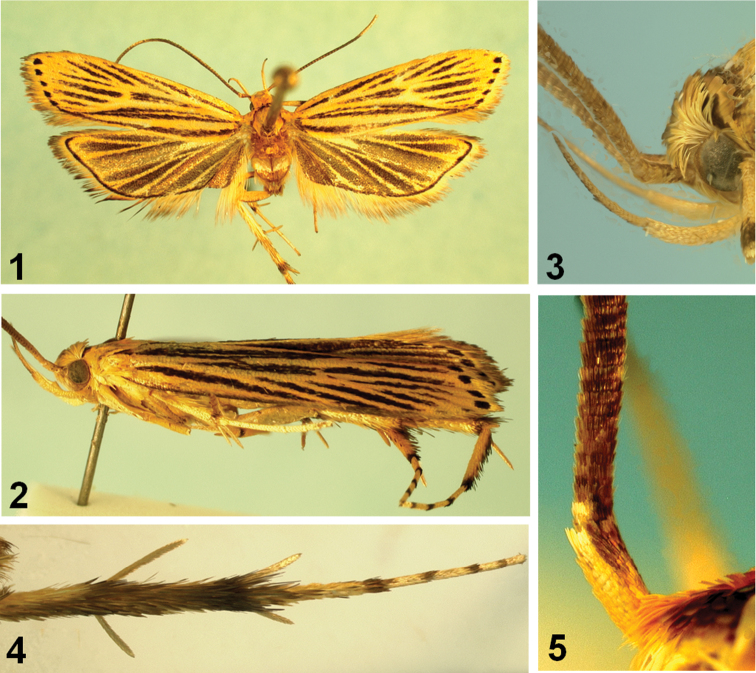
Adult of *Caveana senuri* Park, sp. n. **1** adult, paratype **2** ditto, lateral view **3** labial palpus **4** hind tibia **5** basal part of antenna.

Male and female (Figs 1–5, 11): Wingspan, 17–18 mm. Head light orange. Basal segment of antenna (Fig. 5) elongate, light orange, speckled with brownish scales dorsally; flagellum dark brown, sometimes paler from near half to before 7^th^. Second segment of labial palpus (Fig. 3) gently arched, shiny pale orange; 3^rd^ segment slender, as long as 2^nd^ segment, pale orange speckled with dark-brown scales, with acute apex. Thorax and tegula light orange. Forewing elongate; ground color light orange, clothed with dark-brown scales between veins; costa nearly straight, then gently arched beyond ¾, blackish along anterior margin; apex obtuse; 5-6 large, blackish spots from apex to tornus along termen; termen oblique, not sinuate; fringe light orange in basal 1/3, then dark brown; venation (Fig. 11) with R_1_ arising from before middle, R_2_ arising near upper corner of cell, R_3_ and R_4_ stalked near 2/3 length of R_3+4+5_, R_4_ and R_5_ stalked about 3/5 length; R_5_ reach before apex, M_1_ close to R_3_ at base, M_2_ straightly extended from lower margin of discal cell, M_3_ on common stalk with CuA_1+2_, CuA_1_ and CuA_2_ stalked beyond middle. Hindwing evenly clothed with dark-brown scales, except on veins; light orange along veins; distinct blackish line well-developed from prior to apex to tornus along margin; venation with Rs and M_1_ nearly connate, M_2_ absent, M_3_ and CuA_1_ stalked. Fore and mid tibia with black scales at apex. Hind tibia (Fig. 11) with rough, dark-brown scales above, denser near apex; tarsi with black scales at apex on each segment. Abdomen clothed with dark-brown scales; abdominal tergites with dense spines; sternite VIII bilobed medially, as indicated in Fig. 12.

**Figures 6–12.**
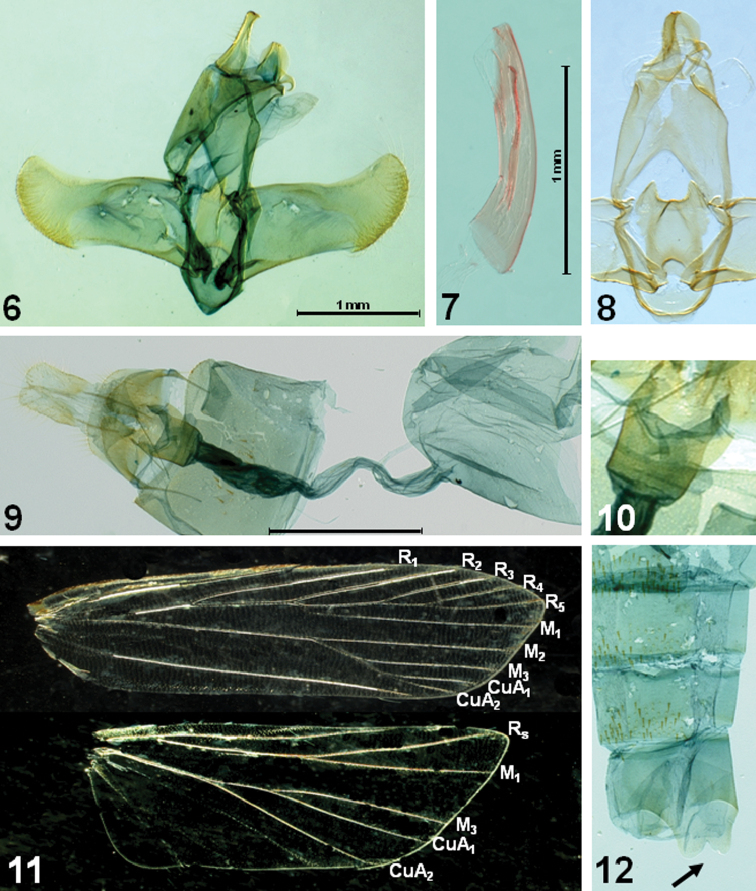
Genitalia and wing venation of *Caveana senuri* Park, sp. n. **6** male genitalia **7** aedeagus **8** juxta and vinculum **9** female genitalia **10** ditto, close-up of antrum; **11** wing venation **12** abdominal segment (arrow indicates the bilobed sternite VIII). Scale bar: 1 mm.

*Male genitalia* (Figs 6–8). Uncus elongate, heavily sclerotized, broadened basally; apex slightly bifurcate. Gnathos relatively short, small, strongly bent downward beyond 2/3. Tegumen long, relatively broad; anterior margin deeply concave. Valva broad; costa slightly concave beyond middle, nearly parallel to ventral margin; cucullus short; outer margin rounded, with dense setae along margin. Juxta shield-shaped, concave in U-shape on caudal margin, with triangular caudal lobes laterally; anterior margin deeply concave. Vinculum narrow, band-shaped. Saccus short, rounded. Aedeagus rather slender, as long as valva, slightly bent; cornutus long, narrow sclerite, as long as 2/3 length of aedeagus. Abdominal tergites with dense spinose zones; sternite VIII bifurcated medially (arrow indicated in Fig. 12).

*Female genitalia* (Figs 9, 10). Abdominal sternite VIII weakly sclerotized, deeply emarginated on caudal margin medially. Apophyses anteriores less than half length of apophyses posteriores. Antrum (Fig. 10) cup-shaped, weakly sclerotized, about 1/4 length of ductus bursae. Ductus bursae longer than corpus bursae, broadened in distal 2/5 length, then slightly narrowed; ductus seminalis arising from near conjunction with corpus bursae. Corpus bursae large, ovate; signum absent.

#### Holotype.

♂, Taiwan, Kaohsiung County, Lukuei Forest Station., 750 m, 29 iv- 3 v 1989 (J. Heppner & H. Wang), deposited in MCUF.

#### Paratypes.

1♂, 1♀, same data as the holotype, genitalia slide no. CIS-6138/Park (♂), -6139/Park(♀); 1♀, Taiwan, Nantou Co., Lu-shan, 30 km E Wushe, 1000 m, 27–31 v 1980 (D.R. Davis), gen. slide no. USNM-92404; 1 gen. slide no. CIS-6138/Park; 1♂, Taiwan, Nantou Co., 15 km E of Puli, 700 m, 6 v 1989 (J. Heppner & H. Wang).

#### Distribution.

Taiwan.

#### Etymology.

The specific epithet is a Korean term, *senuri*, meaning “a new country”.

### Lecithocera dondavisi

Park sp. n.

urn:lsid:zoobank.org:act:6D63A8D5-E85E-4D8E-9F14-95ED992901E6

http://species-id.net/wiki/Lecithocera_dondavisi

Figs 13–23

#### Diagnosis.

This species is one of the largest species of *Lecithocera*. It is externally similar to *Lecithocera praeses* Meyrick, 1919 from North India, but can be distinguished by different following genital features: male genitalia with uniquely specialized cornuti of aedeagus, consisting of a heavily sclerotized ellipticity with an acute spine apically, a heavily sclerotized, elongate trapezoidal plate, and a series of spines, as in Figs 17, 18; and also cucullus with more gently arched ventral margin and juxta not so much produced latero-caudally. Female genitalia with cup-shaped antrum, instead of the elongate, more or less triangular antrum in *Lecithocera praeses*, and the signum strawberry-shaped, located medially, whereas it is transverse elongated and located posteriorly in the latter.

#### Description.

**Figures 13–23.**
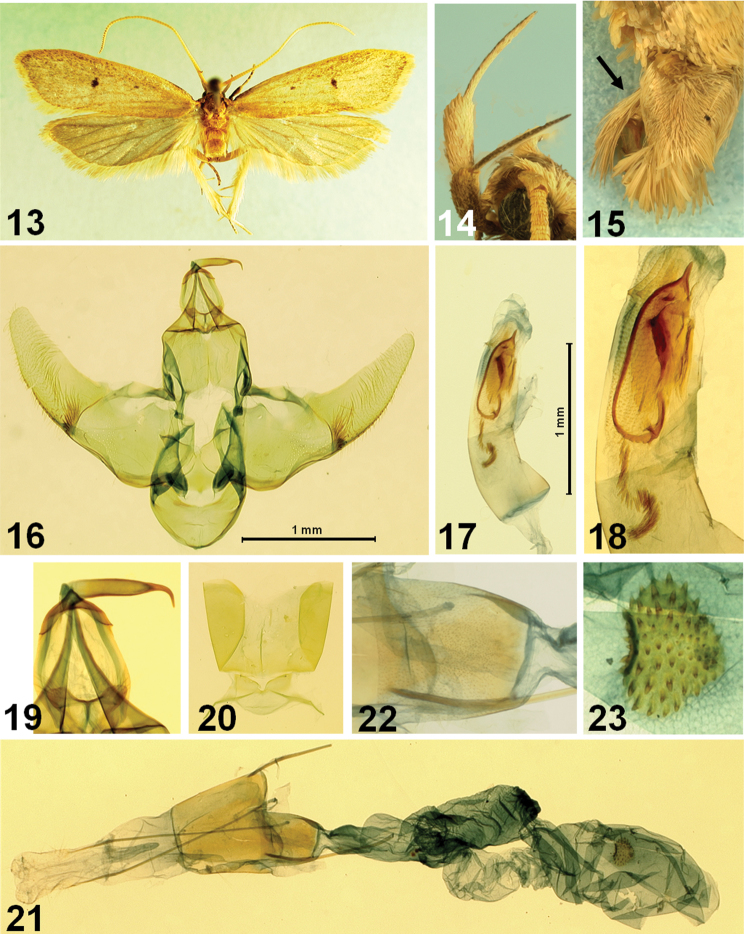
*Lecithocera dondavisi* Park, sp. n. **13** adult, paratype **14** labial palpus **15** terminal segments of abdomen (arrow indicates the dorsal scale-tuft) **16** male genitalia **17** aedeagus **18** close-up of cornuti **19** close-up of signum **20** abdominal sternite VIII **21** female genitalia **22** close-up of antrum **23** close-up of signum. Scale bar: 1 mm.

Male and female (Figs 13–15). Wingspan, 23–26 mm. Head yellowish brown medially on vertex, with pale grayish-orange erect scales laterally; frons pale grayish-orange. Basal segment of antenna rather short, pale grayish orange; flagellum orange white to pale grayish–orange, with distinct brownish annulations in apical third. Second segment of labial palpus (Fig. 14) thickened with appressed scales, grayish orange on outer surface, speckled with dark-brown scales in basal 2/3, orange white to pale grayish orange on inner surface; 3^rd^ segment slender, shorter than 2^nd^ segment, dark brown on ventral surface, with acute apex. Thorax and tegula yellowish brown. Forewing elongate; ground color pale grayish orange, speckled with fine dark-brown scales, more dense posteriorly; first discal stigma small, dark brown at middle of cell; second stigma larger, dark brown, at end of cell; basal blackish streak running along costa in ¼ length; costa nearly straight, then gently arched beyond ¾; apex obtuse; termen oblique, not sinuate, dark-brown scales along margin; fringe orange white in basal 1/3, then brownish; venation with R_1_ arising from before middle, R_2_ arising near upper corner of cell, distance between R_1_ and R_2_ about 2.5 times of distance between R_2_ and R_3_; R_2_ free; R_3_ and R_4_ stalked near middle; R_5_ reach apex; M_1_ at middle between R_3_ and M_2_, M_2_ nearly parallel with M_1_; M_3_ at middle between M_2_ and CuA_1+2_; CuA_1_ and CuA_2_ very short-stalked. Hindwing pale gray, broader than forewing; apex obtuse; termen oblique, slightly sinuate; fringe grayish, with orange white basal line; venation with, M_2_ well developed, connected to M_3_ with cross vein; M_3_ and CuA_1_ short-stalked; cell nearly closed with an oblique cross vein. Hind tibia with orange-white rough scales above. Abdomen with pale grayish-orange scales dorsally, with a well-developed scales-tuft dorsally in terminal segment, as indicated in Fig. 15; sternite VIII bilobed medially, as indicated in the Fig. 12.

*Male genitalia* (Figs 16–22). Basal lobes of uncus more or less semiovate, gently concave on caudal margin. Gnathos (Fig. 19) relatively slender; apical part heavily sclerotized, strongly bent downward. Tegumen weakly sclerotized with anterior margin incised medially. Valva broad at base, width as wide as length of tegumen; costal bar connecting with tegumen strong, angled medially; ventral margin gently concave before cucullus; cucullus elongate, narrowed towards apex, dense setose, with bundle of setae at lower corner at base, apex rounded; sacculus sclerotized, slender. Juxta shield-shaped, with small projection at middle on anterior margin; caudal margin slightly emarginated, with crescent extension laterally. Vinculum broad, with round apex. Saccus round. Aedeagus (Figs 17, 18) with uniquely specialized cornuti of aedeagus, consisting as heavily sclerotized ellipticity with acute spine at apex, about half length of aedeagus, and a row of short spines. Abdominal tergites without spines; sternite VII-VIII as figured in Fig. 20.

*Female genitalia* (Figs 21–23). Abdominal sternite VIII weakly sclerotized, nearly straight anterior margin. Apophyses anteriores thick, short, nearly 1/5 length of apophyses posteriors. Antrum (Fig. 22) cup-shaped, weakly sclerotized, about 2/3 length of abdominal sternite VIII. Ductus bursae slightly longer than corpus bursae, shortly necked between antrum and ductus bursae, then broadened; ductus seminalis as broad as ductus bursae, arising from middle. Corpus bursae large, elongate; signum strawberry-shaped, with dense conic spines.

#### Holotype

**.** ♂, Taiwan, Hsinchu County., Kuangwu, 24-25 vi 1985 (J. Heppner & H. Wang), gen. slide no. CIS-6168/Park, deposited in MCUF.

#### Paratypes.

4 ♂, 1♀, same data as the holotype, gen. slide no. CIS- 6192/Park(♀); 1♂, Taiwan, Nantou Co., Meifeng 30 km S Tayuling 2200 m, 1-8 vi 1980 (D. R. Davis), gen. slide no. USNM-92499/Park.

#### Distribution.

Taiwan.

#### Etymology.

The species is named after Dr. Donald R. Davis, Curator of Lepidoptera, US National Museum Natural History, Smithsonian Institution, USA, an authority on the microlepidoptera of the world.

## Supplementary Material

XML Treatment for Caveana senuri

XML Treatment for Lecithocera dondavisi
